# Community facilitators and barriers to a successful implementation of mass drug administration and indoor residual spraying for malaria prevention in Uganda: a qualitative study

**DOI:** 10.1186/s12936-018-2624-7

**Published:** 2018-12-17

**Authors:** Humphrey Wanzira, Susan Naiga, Ronald Mulebeke, Fred Bukenya, Mariam Nabukenya, Osborn Omoding, Dorothy Echodu, Adoke Yeka

**Affiliations:** 1 Pilgrim Africa, Kampala, Uganda; 2 Infectious Diseases Research Collaboration, Kampala, Uganda; 3 Ministry of Health, National Malaria Control Program, Kampala, Uganda; 4 Makerere University School of Public Health, Kampala, Uganda

**Keywords:** Mass drug administration, Indoor residual spraying, Malaria

## Abstract

**Background:**

There is growing interest to add mass drug administration (MDA) to the already existing malaria prevention strategies, such as indoor residual spraying (IRS). However, successful MDA and IRS requires high population-wide coverage, emphasizing the importance of community acceptance. This study’s objectives were to identify community-level facilitators and barriers during the implementation of both MDA and IRS in communities with high malaria transmission intensity.

**Methods:**

This was a qualitative study conducted in two sub-counties in Katakwi district. Kapujan sub-county residents received two rounds of IRS and MDA while Toroma sub-county residents received two rounds of IRS only. Key informant interviews and focus group discussions were conducted with key influential district and sub-county personnel and community members. Data were analysed using thematic analysis. Transcripts and interview notes from the in-depth interviews were analysed using a coding scheme developed from pre-defined topics together with themes emerging from the data. The Nvivo software program was used to aggregate the data by codes and to present study findings.

**Results:**

Overall, 14 key informants were interviewed: 4 from Katakwi district and 5 each from Kapujan and Toroma sub-counties. Five focus group discussions were conducted: 4 with community members (men and women), 2 in each sub-county and one with medical staff of Toroma health centre IV. Important themes for consideration raised by the respondents include community sensitization, conducting implementation during the low activity dry season, involvement of government and local leadership, use of the competent locally composed team, community knowledge of malaria effects and consequences, combining interventions and evidence of malaria reduction from interventions. Potential barriers such as spreading of misinformation regarding interventions, the strong unpleasant smell from Actellic and inadequate duration of engagement with the community should be taken into consideration.

**Conclusion:**

This study documents important community engagement strategies that need to be considered when implementing malaria MDA in combination with IRS, for malaria prevention in such settings. This information is useful for malaria programmes, especially during the design and implementation of such community level interventions.

## Background

In Uganda, malaria is endemic in approximately 95% of the country and affects over 90% of the population. It is responsible for 30 to 50% of out-patient admissions, 15 to 20% of in-patient admissions and 20% of hospital deaths [1]. Pregnant women and children under 5 years old are the most affected. Additionally, findings from the 2014 Malaria Indicator Survey (MIS) estimate malaria prevalence among children under 5 years at 19% [2]. Malaria is therefore considered a disease of public health importance by the National Malaria Control Programme (NMCP) which has implemented a number of interventions to reduce this burden, including the distribution of long-lasting insecticidal nets (LLINs), indoor residual spraying (IRS), intermittent preventive therapy (IPTp) in pregnant women, and malaria case diagnosis with prompt treatment [1].

However, there is evidence to suggest that vector control and malaria case management may not be adequate to control malaria and reduce transmission in all areas [3]. Consequently, there is growing interest in treating asymptomatic individuals to augment malaria control and reduce malaria transmission, and in targeting hotspots of malaria transmission to reduce the human infectious reservoir [4]. One of the treatment approaches considered is mass drug administration (MDA), others are intermittent preventive therapy (IPT) and intermittent screening and testing (IST) [4, 5]. MDA is the empiric administration of a therapeutic anti-malarial regimen to an entire population at the same time. The re-emergence of MDA as a potential intervention especially for malaria elimination is because of the availability of safe, efficacious and long-acting artemisinin-based combination therapy (ACT), such as dihydroartemisinin–piperaquine (DP) [6–8]. Indeed, trials conducted in the other settings have shown MDA using DP to be successful in reducing population parasite prevalence. For instance, in Cambodia, MDA given once, or in some cases repeated at day 42, to the entire population successfully reduced the parasite prevalence from 52.3 to 2.6% after 3 years [9].

The processes of carrying out an MDA programme are complex, especially in large communities. Evidence from other MDA studies of disease conditions such as lymphatic filariasis [10, 11] and schistosomiasis [12] have shown that community involvement is essential for the success of such campaigns. However, the high burden of malaria and the high transmission rates, coupled with multiple and sometimes complex treatment protocols [1], may require a different or robust community engagement approach beyond what is already known or done for other disease conditions. This could be further complicated when MDA is implemented in combination with other vector control strategies, such as IRS. The importance of high population coverage of such interventions is essential for their success, emphasizing the importance of community acceptance [13, 14]. A recent mathematical modelling publication by Brady et al. demonstrated that the higher the effective coverage of an intervention, the greater its impact [15]. Therefore, understanding the community’s dynamics and perceptions is one of the important steps towards this goal. This study set out to identify community-level facilitators and barriers for the implementation of both MDA and IRS at community level.

## Methods

### Study design

This is a qualitative study that was conducted between April and June 2017 and reported according to the standards of reporting of qualitative research [16]. It is part of a larger quasi-experimental project conducted in Katakwi district, an area of high malaria transmission intensity. According to baseline malaria data obtained from the 2016 Health Management and Information System (HMIS), the three sub-counties reported 4037 laboratory-confirmed malaria cases of the total tests done, which translated to a 30% positivity rate [17].

### Summary description of the intervention quasi-experimental study

This is an ongoing project that commenced in August 2016 with the primary objective to investigate the impact of population-based MDA in combination with IRS on both clinical and entomological malaria indicators. It is conducted in three sub-counties of Katakwi district: Kapujan, Toroma and Magoro whose population estimates are 14,468, 11,825 and 18,564, respectively. At baseline, all three sub-counties received LLINs as part of the 2013 and 2017 universal distribution campaigns, however, none received any IRS. This study intervention is MDA using DP, and IRS using Actellic, implemented as follows: Kapujan households and residents receive both IRS and MDA, Toroma households receive IRS only, while Magoro is the control sub-county and continues to receive the standard of care malaria prevention and treatment activities, such as universal distribution of LLINs, IPTp, diagnosis of malaria and prompt treatment with ACT [1]. Specific to the study, interventions are implemented at the same time following approximately a 6-monthly interval, with the first round conducted in December 2016. Before intervention implementation, community-wide information, education and communication (IEC) was conducted to raise awareness of the study. National and sub-national stakeholders, including the district and sub-county leadership, were engaged to be part of the IEC team. Informed consent was sought from household heads before IRS implementation and all individuals eligible to take MDA. Specifically, for MDA, additional assent was sought from children between 8 and 17 years before their guardian’s consent on their behalf. The guardians of children aged 6 months to 7 years also consented on their behalf. To minimize contamination among the sub-counties, the following was done: group-level interventions such as assigning the whole sub-county to intervention with all households (for IRS) and members (for MDA) eligible for intervention; before the start of interventions, all households (using global position system for location and to demarcate sub-county boundaries) and household members were enumerated and households were assigned a unique ID placed on the household front door while members receiving MDA had a unique ID bar code placed on participant’s exercise book; at the time of intervention, only households and members assigned a unique ID received intervention; all sub-county members were eligible to be sampled for measurement of effect of the interventions. Preliminary results from the first round show that the coverage for MDA was 80.6% (13,353 out of 16,577 individuals registered) while IRS coverage was estimated 98.4% (11,662 out of total 11,851 structures found).

### Study setting, population and sampling strategy

This qualitative study was conducted in Kapujan and Toroma sub-counties, for which intervention was implemented. Participants for the key informant interviews (KII) were selected from various levels of the district administration, community and religious leadership after consultation with district and sub-county leaders and relevant stakeholders. They were selected purposively based on their engagement with study personnel and virtue of their position in the district, which gave them insight on people’s experiences with the study interventions. They were first contacted by phone to inform them of the interviews and to set an interview time. Focus group discussions (FGDs) comprised both men and women from communities in the two sub-counties. One FGD was conducted with health facility staff of the largest health centre IV that covers both sub-counties. Sub-county and village leaders were contacted and asked to suggest members to be included in the FGDs. Each focus group comprised 8–12 members.

### Data collection tool and study procedures

Semi-structured KIIs and FGDs interview topic guides were developed and pre-tested for reliability. Topics covered included: malaria prevention and interventions in the community; conduct of MDA and/or IRS; practicality of implementation; and, adaptation and integration while probing for facilitators and barriers. Two trained and experienced personnel were involved during the interview process, an interviewer and an assistant who took notes of the discussion. All interviews were also recorded using a digital voice recorder. Written informed consent was sought before and all interviews were conducted in a language that was most comfortable for the interviewee, either in English or Ateso, the locally spoken language. Summaries of the interviews were all written in the language of the interview, and later translated into English. All these activities were supervised by two experienced IEC study team members and one qualified qualitative study expert with extensive experience in conducting such studies.

### Data processing and analysis

Data were analysed using thematic analysis. Transcripts and interview notes from the in-depth interviews were analysed using a coding scheme developed from pre-defined topics together with themes emerging from the data. Analysis was conducted independently by different members of the study personnel on different transcripts and then a final coding scheme was agreed on and applied to all transcripts, with at least two members of the study personnel reviewing each transcript. The reviewers went through the transcripts, discussing and agreeing how best to code the transcripts while reviewing the existing codes. The Nvivo software program was used to aggregate the data by codes and to assist with findings presentation.

## Results

### Characteristics of study participants

Fourteen key informants were interviewed: 4 from Katakwi district and 5 each from Kapujan and Toroma sub-counties (Table 1). Five FGDs were conducted: 4 with community members (men and women), 2 in each sub-county and one with medical staff of Toroma health centre IV.

**Table 1:** Key informant respondents

Key informant	District level, N = 4	Sub-county level	
		Kapujan, N = 5	Toroma, N = 5
District health officer	1	–	–
Local council V chairman	1	–	–
Malaria focal person	1	–	–
Health secretary	1	–	–
Local council III chairman	–	1	1
Health centre IV in-charge	–	–	1
Manager private provider clinic	–	1	–
Opinion leader	–	1	1
Religious leader	–	1	–
School head teacher	–	1	1
Village health team	–	–	1

### Emerging themes

#### Facilitators for intervention

There were 10 main facilitators to the intervention which included:

#### Informed consent for community leadership and members before intervention

All respondents (key informants and from FGDs) reported having consented for both IRS to be applied in their household and MDA for individuals after getting adequate information regarding these interventions before they were implemented. Additionally, the district health officer and local council leaders also said that they had been contacted to give consent for the interventions to be conducted in their areas.

*“Before someone makes an informed consent, that person would have gotten information. They should point out the benefits, the pros and cons, the adverse effects so that even the lay man in the village will know”* FGD health workers

#### Intervention conducted in the dry season

Two FGD respondents felt that the interventions had been implemented at the right time when the mosquitoes were many, the season was dry and people were harvesting, which meant they were more available at home to be involved in study-related activities.

#### Community sensitization and mobilization of relevant stakeholders

Four influential key informants, including the district health officer, district secretary for health, two local council chairmen (levels III and V) and respondents of one FGD mentioned that sensitization and mobilization of all relevant stakeholders, including the community and the local leaders, for the interventions was being handled well by the study team.

*“At the onset, there was mobilization and advising people on how the interventions were to be conducted. So if people were not sensitized, this project would not have been a success. Because if you want to do anything, you must tell the person its advantages and disadvantages so when people understand that, it is very easy for them to accept.”* LC III chairman

For IRS specifically, the FGD respondents were happy that the community mobilizers went to their households ahead of the spraying team, telling people to move their belongings out of the houses before the spraying team arrived.

#### Technically competent and trusted implementation team

Respondents from all the FGDs and two key informants mentioned that they appreciated several attributes of the team that implemented the interventions, such as their friendly attitude, focus on their work, punctuality, approachable, knowledgeable and consideration:

*“The team, they were knowledgeable on how they mixed the spray products and prescribed the drugs. I think they were mixing it in a correct way and they could tell you to cover your things before, which was a sign of being kind other than just working. In case there was some things which were big and you couldn’t carry out, they were helpful.”* FGD respondent

#### Local implementing team members

Respondents from two FGDs mentioned that having the implementing team members employed from the local area played a big role in successful interventions implementation, especially IRS:

*“Since the spray operators were from within, they could even just ride bicycles or sometimes they could even go on foot. To us this was the best, our own community members were selected and as a result they worked with one heart because they knew what their community was going through as a result of the burden of malaria.”* FGD medical staff respondent

#### Government involvement

All key informants and respondents from three FDGs believed that the implementing organization had sought permission from government to implement the interventions and as such they felt confident to participate in the interventions. Also many of the respondents participated because they had confidence in the authenticity of the organization and its activities.

#### Involvement of local leadership

All the key informants and respondents from FGDs cited several community factors that supported the interventions, such as local involvement of key influencers including the local leaders. For example, the village health team members helped with follow-up of participants during the MDA exercise by visiting homes to ensure adherence.

*“… the local leadership and government should take a step in educating its people because that is their role. Secondly religious leaders should also pass on this information. It is the responsibility of these leaders to educate their people.”* FGD community member

#### Community knowledge of the cost of preventing and treating malaria

Six key informants and respondents from FGDs mentioned that they participated in the study because of the high expenses they incur preventing and treating malaria. The knowledge that the communities spend high expenses treating frequent malaria episodes was a motivator for taking up IRS and MDA:

*“I accepted because I used to spend a lot of money treating malaria. I personally often fell sick almost every 2* *weeks. So when I heard about IRS I knew my problems would be solved.”* FGD community member

#### Evidence of reduced malaria burden after the intervention

All key informant interviewees and respondents from all FGDs mentioned that they had observed a drastic drop in malaria cases in the areas where intervention was being conducted. They also got study updates as part of the Community Advisory Board. Additionally, health-worker FGD respondents also observed a drastic drop in malaria cases especially those stationed at the out-patient department (OPD), which prompted them to not only accept the interventions but also to encourage the community to take them up.

*“When you actually see the attendance of the OPD register, malaria has reduced, basically possibly because of indoor residual spraying. It has reduced the workload of OPD, you find that patients are now very few.”* Health facility health worker

#### Setting up centres to manage drug side effects

Respondents from three FGDs mentioned that putting in place a system of managing side effects at the local health facilities strengthens community confidence in the intervention and encourages participation.

*“In my opinion, I was of a view that if the organization is well equipped, let there be nurses to attend to emergency cases that arise as a result of MDA. Let them be put in various health units to help us with such cases because in most cases the nurses who are in these health centres already have their work load and as a result, they tend to ignore us when we reach the units. One thing I know is that the drug cannot be brought knowing it can harm…”* FGD community member

#### Combining IRS and MDA interventions for malaria prevention

The district malaria focal person, a religious leader and FGD respondents from the sub-county that was receiving IRS only suggested that instead of receiving one intervention (IRS only), they should also be included among those receiving MDA because this would enhance malaria prevention in their community, an important aspect for their continued engagement.

*“Spraying alone can’t help so to me let all the three interventions be brought together, e.g. MDA, IRS and the mosquito nets. Right now, yes, our houses have been sprayed but when we sit outside sometimes mosquitoes still bite us before bed*-*time. So if there was medicine I am sure it would work best.”* FGD community member

#### Challenges to intervention

Three challenges included:

#### Instances of community mis-information that lead to the fear of taking up intervention

This was mentioned by respondents from four FGDs conducted in the areas that were receiving both interventions. There were instances of mis-information regarding the intervention mainly from community individuals who did not understand the interventions or had opposing views to either MDA or IRS. Some of this mis-information caused fear among community members that lead to a few refusing the intervention:

*“I remember of the case where some people refused to take the drug. Something that actually happens and results in people being mislead. That these drugs can cause some effects for example they said that these drugs will make men fail to perform in bed. But then to some of us that was not the issue because we swallowed and there was no problem.”* FGD community member

#### Household member inconvenience during IRS implementation

Respondents from the four FGDs conducted in the IRS sub-counties described the spraying exercise as cumbersome especially for households that possessed a lot of property in their homes. This is because the process required moving everything out of the house to allow for the spray to be conducted. Additionally, some respondents complained about the smell of Actellic after the spraying of their households.

*“People were complaining about the smell of the drug that was used for spraying houses because it was causing uneasiness, others say it caused vomiting, others said I got diarrhea yesterday because of the smell of the drug.”* Key informant village health team member

#### Short time allocated for implementation of intervention

A few respondents from two FGDs felt that the time allocated for IRS and MDA was not enough with some community members observing that the implementation teams were in a hurry to complete the work.

## Discussion

This study documents important facilitators and barriers to be considered for a successful community-wide implementation of both MDA and IRS for malaria prevention in a high malaria transmission setting. For instance, community sensitization and mobilization campaigns were recognized as an important activity for a successful intervention uptake. This finding is supported by similar studies conducted in The Gambia and Cambodia which showed that such sensitizations are crucial for community adherence to MDA [18]. These are similar findings reported from MDA studies conducted for conditions such as lymphatic filariasis [10, 11]. In this study, a number of communication channels were used, including radio announcements and talk shows, village meetings and information posters. Information such as the description of interventions, their importance, frequency of implementation, recipients, risks and benefits was included. This was further complimented by person-to-person interactions before any interventions could be implemented, especially regarding informed consent procedures. This sensitization campaign could have been the most important strategy responsible for the observed high population coverage of this study [19–21]. Additionally, the use of already existing health facility infrastructure and personnel as centres to manage any occurring MDA and IRS adverse events could have possibly provided additional confidence for the community to take up the intervention.

It was also noted that the more knowledgeable individuals were about the effects and consequences of malaria, the more they were willing to take up MDA and IRS. This is because of the anticipated advantages these interventions would bring to their families and communities [22–24]. This finding lends support to the importance of IEC and behaviour change communication (BCC) prior to intervention implementation. This was further strengthened when community leadership and health workers observed evidence of reduced number of mosquitoes and clinical malaria burden in their communities following intervention implementation, which is a classic example of proof of intervention effectiveness. Furthermore, some residents especially in the IRS-only sub-county were advocating having MDA in their area, in addition to IRS, because this was known to work synergistically to reduce the malaria burden.

The importance of involving key government institutions, such as NMCP and sub-national levels during community-wide interventions was echoed by almost all of the key informants. Greater community assurance is attached to interventions for which the implementing partners collaborate with the leadership [18, 22, 24]. In this study, this collaborative involvement with government institutions was not only at central level, but included district, sub-counties and village levels. Other important facilitators for improvement of community confidence included a trusted implementing partner who is known by the community to deliver significant interventions, and the use of a technically competent team mostly made up of local community members who are familiar with the population and setting [24].

The majority of community members appreciated the timing of the intervention during the dry harvesting season. This is traditionally a less busy working season for this mainly agricultural community because most household members are engaged in crop harvesting and were, therefore, available at home with time to be engaged in other activities. This is crucial, especially for the MDA intervention, which requires individual household members to be present so as to take the medication. Indeed, there is evidence from another qualitative MDA study from The Gambia showing that conducting such exercises, especially in rural settings, required a time/season when individuals were less engaged in their farmlands and available at home [17].

However, there were a few barriers that were mentioned by a few respondents that still warrant recognition so as to minimize and prevent them from worsening during such community campaigns. Such challenges included instances of community misconceptions and mis-information by individuals who either do not understand the interventions or had opposing views. This sometimes led to fear among some community members who opted not to take up the interventions. Such instances have been reported in other studies conducted in Asia and other African settings that showed the negative impact of community misconceptions on MDA acceptability [18, 25, 26]. Specific to IRS, the strong unpleasant smell of Actellic has been well documented [27, 28] and there is the possibility that this could be a deterring issue for uptake of this intervention when using this compound. Finally, the duration of intervention implementation should be longer to allow the community greater and adequate interaction with the implementation team. This would enhance community knowledge and acceptance of intervention.

There are study limitations that need to be acknowledged, such as the small sample size which could be argued to affect the validity of these findings. There is also the possibility of researcher bias from the purposive sampling design of the key informants, since their responses are personal impressions. However, both of these instances were minimized by triangulating information from two sources of data collection: key informant interviews and FGDs. Additionally, there was extensive consultation undertaken to include important and influential personnel from the different levels of leadership for key informant interviews, while community village leaders were part of the selection of focus group members.

## Conclusion

Community sensitization on interventions, including malaria-specific knowledge, implementing interventions during the low farm-related activity periods, involvement of government and local leadership during implementation, and the use of a competent implementation team are essential for a high population acceptance of both MDA and IRS in this community. Potential barriers, such as spreading of mis-information regarding interventions, the strong unpleasant smell from Actellic, and inadequate duration for engagement with the community should be taken into consideration to limit their negative impact. Even though most of these findings are similar to those from other MDA or IRS from other settings, this study adds to literature by documenting community engagement strategies when implementing these interventions in combination, in such settings.

## Abbreviations

ACT: artemisinin-based combination therapy
BCC: behaviour change communication
DP: dihydroartemisinin–piperaquine
FGDs: focus group discussions
HDREC: Health Higher Degrees Research and Ethics Committee
HMIS: Health Management and Information System
IEC: information, education and communication
IPT: intermittent preventive therapy
IRS: indoor residual spraying
KIIs: key informant interviews
LC: local council
LLIN: long-lasting insecticidal bed nets
MDA: mass drug administration
MIS: Malaria Indicator Survey
NMCP: National Malaria Control Programme
OPD: out-patient department
UNCST: Uganda National Council for Science and Technology
